# Supplementary material for: Vitamin D Modified DSS-Induced Colitis in Mice via STING Signaling Pathway
Source: Biology (Basel). 2025 Jun 18;14(6):715. doi: 10.3390/biology14060715 (PMC12190092; doi:10.3390/biology14060715)
Supplement: Supplementary file 1 [file biology-14-00715-s001.zip › Supplementary Materials S1: 16s-report of gut microbiota/Groups/diff_analysis/metagenomeRGMM/figure/barplot/top10.pdf]

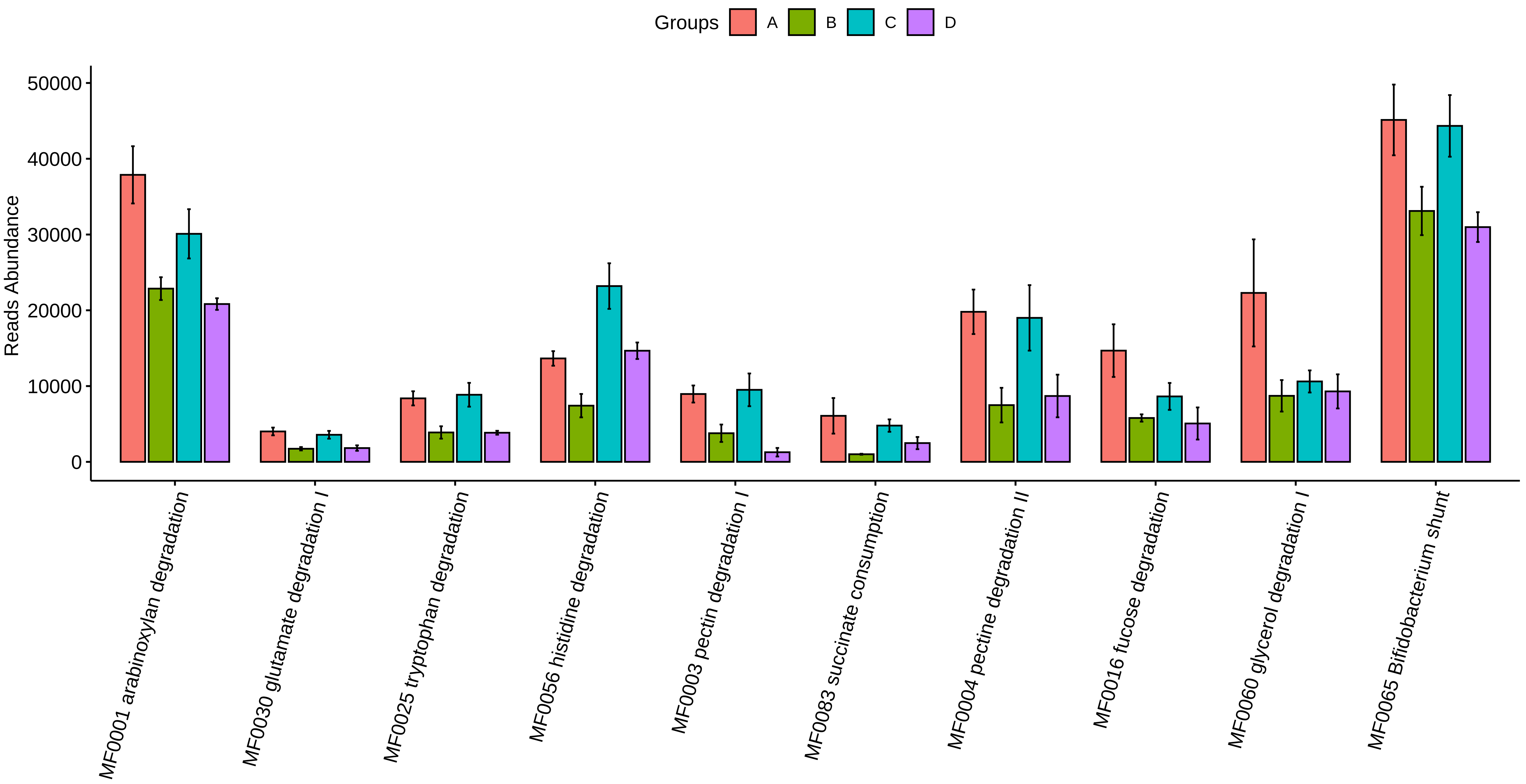
Groups
A
B
C
D
Reads Abundance
50000
40000
30000
20000
10000
0
MF0001 arabinoxylan degradation
MF0030 glutamate degradation I
MF0025 tryptophan degradation
MF0056 histidine degradation
MF0003 pectin degradation I
MF0083 succinate consumption
MF0004 pectine degradation II
MF0016 fucose degradation
MF0060 glycerol degradation I
MF0065 Bifidobacterium shunt
